# Voxel-Based Statistical Analysis of 3D Immunostained Tissue Imaging

**DOI:** 10.3389/fnins.2018.00754

**Published:** 2018-11-15

**Authors:** Michel E. Vandenberghe, Nicolas Souedet, Anne-Sophie Hérard, Anne-Marie Ayral, Florent Letronne, Yaël Balbastre, Elmahdi Sadouni, Philippe Hantraye, Marc Dhenain, Frédérique Frouin, Jean-Charles Lambert, Thierry Delzescaux

**Affiliations:** ^1^CEA, DRF, Institut François Jacob Molecular Imaging Research Center, Fontenay-aux-Roses, France; ^2^Neurodegenerative Diseases Laboratory, CNRS, CEA, Paris-Sud University, Paris-Saclay University UMR9199, Fontenay-aux-Roses, France; ^3^INSERM U1167, Institut Pasteur de Lille Université Lille-Nord de France, Lille, France; ^4^Laboratoire Imagerie Moléculaire in vivo (IMIV UMR 1023 Inserm/CEA/Université Paris Sud - ERL 9218 CNRS) Orsay, France

**Keywords:** voxel-based, 3D, immunohistochemistry, microscopy, rodent, Alzheimer's disease

## Abstract

Recently developed techniques to visualize immunostained tissues in 3D and in large samples have expanded the scope of microscopic investigations at the level of the whole brain. Here, we propose to adapt voxel-based statistical analysis to 3D high-resolution images of the immunostained rodent brain. The proposed approach was first validated with a simulation dataset with known cluster locations. Then, it was applied to characterize the effect of ADAM30, a gene involved in the metabolism of the amyloid precursor protein, in a mouse model of Alzheimer's disease. This work introduces voxel-based analysis of 3D immunostained microscopic brain images and, therefore, opens the door to localized whole-brain exploratory investigation of pathological markers and cellular alterations.

## 1. Introduction

Microscopic investigation of brain tissue architecture and composition plays an essential role in understanding the mechanisms underpinning brain function and disease. Classically, the brain microstructure is studied *post mortem* via histological processing. However, this results in the loss of brain 3D structure. Recently, a wealth of techniques have been proposed for optical 3D microscopic imaging of the entire rodent brain, or large portions thereof. Proposed techniques fall into three main categories: tissue clarification followed by light sheet microscopy (Dodt et al., 2007; Ertürk et al., 2012; Chung et al., 2013; Ke et al., 2013; Susaki et al., 2014; Hama et al., 2015; Renier et al., 2016), serial optical tomography (Ragan et al., 2012; Wang et al., 2014; Wu et al., 2014) and 3D histology (Ourselin et al., 2001; Lein et al., 2007; Grand'maison et al., 2013; Vandenberghe et al., 2016). Already, these techniques have been useful to describe the mouse brain gene expression spatial distribution (Lein et al., 2007), cyto-architecture (Silvestri et al., 2015), connectome (Oh et al., 2014; Zingg et al., 2014) and functional activation patterns (Vousden et al., 2015). Besides neuroanatomical studies, a promising application of 3D microscopic imaging is to quantitatively analyze immunostained markers in experimental studies. Immunohistochemistry (IHC) is the method of choice to reveal the presence of a protein of interest in a tissue sample. Voxel-based analysis of 3D IHC imaging could allow to detect microstructural and cellular modifications induced by experimental conditions, such as genetic interventions or drug administrations. Nevertheless, an important remaining challenge consists in how to infer the location and nature of changes affecting IHC markers between cohorts of animals. In previous works, changes between groups of 3D whole-brain histology images were detected by segmenting the marker of interest and by using a 3D digital brain atlas to delineate regions of interest wherein the marker load was compared between groups (Grand'maison et al., 2013; Vandenberghe et al., 2016). However, this approach supposes that differences between groups can be detected at the regional level which may not hold when the differences are located in very limited areas or when opposite effects take place within the same anatomical region (Dubois et al., 2010).

Voxel-based analysis is an attractive alternative to region-based analysis to detect local differences between sets of brain images (Friston et al., 1990). To our knowledge, voxel-based analysis of 3D IHC imaging has not been achieved yet. Two main challenges prevent the direct application of voxel-based analysis to 3D IHC imaging. Firstly, IHC images are typically captured at a microscopic resolution. At such a high resolution, direct voxel-to-voxel comparisons are pointless because intrinsic variability between subject exceeds the variability that can be reasonably expected from experimental interventions. Secondly, voxel-based analysis applies to continuous signal while IHC staining is a qualitative indicator of the presence of a protein of interest.

Here, we propose a novel approach to extend voxel-based statistical analysis to 3D immunostained brain imaging in rodents. Our approach consists in extracting quantitative signal from high-resolution 3D IHC images and then to summarize it in the form of lower-resolution 3D parametric maps that can be readily compared at the voxel level. We evaluated the accuracy of several methods for parametric map generation using simulation experiments where synthetic segmented IHC images are generated with Boolean-Poisson processes. Voxel-based analysis was then applied in a 3D histology study investigating the impact of the over-expression of the ADAM30 gene on Aβ plaque deposition in a transgenic mouse model of Alzheimer's disease (Letronne et al., 2016). We show that compared to our initial investigation using an region-based approach, voxel-based analysis enables more sensitive and spatially resolved detection of Aβ plaque deposition changes due to ADAM30 over-expression.

## 2. Materials and methods

### 2.1. Datasets

This subsection describes the simulation dataset used for benchmarking and method validation as well as the dataset to study the effect of ADAM30 on the Aβ plaque deposition transgenic mice.

#### 2.1.1. Simulation dataset

Aβ plaques are typically round objects that can vary in size. To account for these properties, simulations were performed with a Boolean-Poisson model, a spatial stochastic process introducing randomness at two levels: object location and object shape (Baddeley et al., 2004). The simulated segmented marker was modeled as disks which radii were drawn from a Gaussian distribution with mean radius *r* = 7 pixels and a standard deviation of 2 pixels. Seven pixels is a typical radius for an Aβ plaque in an image with a pixel-size of 5 μm. Disk centers correspond to the realization of a Poisson point process in two dimensions. Simulation images had a size of 2,560 × 2,560 pixels. Accordingly to the definition of a Poisson point process, the probability of an Aβ plaque being seeded at coordinates *x* of a simulation image is:

(1) $$\begin{array}{r} P\left(\text{seed}\left(x\right)\right)=\lambda \left(x\right)e^{-\lambda \left(x\right)} \end{array}$$

where λ(*x*) is a piecewise-constant intensity function of seeds (and is also a 2D image). Also, μ_*x*_, the true marker load was calculated as the probability that a voxel of an image at location *x* is part of a disk. The mean squared error was calculated between the true marker load and the marker load as estimated from the realizations of the Boolean-Poisson process.

**Figure 1:**
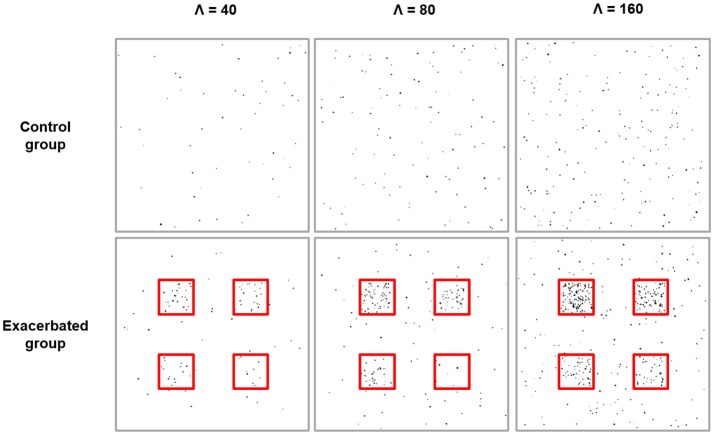
Simulation experiments. From left to right: simulation experiments with basal expected number of disks, Λ, equal to 40, 80, and 160, respectively. For each experiment, one example of a simulation image from the control group is shown at the top and one example of a simulation image from the exacerbated group is shown at the bottom. Disks appear in black and the underlying cluster boundaries are shown in red. For each experiment, the expected relative increase in the number of disks per cluster is either 20 (top left cluster), 10 (top right cluster), 5 (bottom left cluster), or 2.5 (bottom right cluster).

Two types of images were generated to model individuals drawn from a control population and an exacerbated population where disks are more frequent in spatially defined clusters. Generated images were composed of a background with constant rate λ_0_ and four square regions with constant rate λ_1_, λ_2_, λ_3_, λ_4_. In the control group, images were such that λ_0_ = λ_1_ = λ_2_ = λ_3_ = λ_4_, while in the exacerbated group they were such that λ_1_ = 20λ_0_, λ_2_ = 10λ_0_, λ_3_ = 5λ_0_ and λ_4_ = 2.5λ_0_. The control and the exacerbated group consisted of 10 images each, which is a realistic sample size for animal studies. Three different experiments were carried out with various disk basal density conditions (Figure 1). In the first experiment, control group images had a basal rate λ_0_ = 40, which mimics the distribution of Aβ plaques in a relatively low-density condition. In the second and third experiments, rate was increased to λ_0_ = 80 and λ_0_ = 160, respectively.

#### 2.1.2. ADAM30 study dataset

This dataset was previously investigated in Letronne et al. (2016). Two transgenic mouse strains with a C57Bl/6N background (Taconic) were generated either carrying the human ADAM30 gene with a Cre promoter enabling its expression or only the human ADAM30 gene. Cre-ADAM30 mice express ADAM30 conditionally upon Neomycine-mediated Cre activation while the second line does not express the gene because the promoter is absent and, thus, served as negative control to test the effect of ADAM30 on the Aβ load. Cre-ADAM30 and ADAM30 mice were crossed with hAPP_*Swe, Ind*_ mice expressing the human APP gene bearing “Swedish” and “Indiana” mutations which are associated with familial forms of Alzheimer's disease and cause pathological Aβ deposition (Jackson Laboratory) (Mucke et al., 2000). At 10 months of age, 10 APP_*Swe, Ind*_-Cre-ADAM30 and 5 APP_*Swe, Ind*_-ADAM30 were euthanized, the brains were removed from the skull and freshly frozen. For each individual, one hemi-brain was used for 3D histology investigations. All experiments were approved by the local animal care and use committee (Comité d'Ethique en Experimentation Animale du Nord - Pas de Calais, Lille, France).

Hemi-brains were all embedded in a green-colored solid matrix (Neuroscience Associates) to obtain a sharp contrast between the embedding material and the cerebral tissue (Vandenberghe et al., 2016). For each hemi-brain, 100 30-μm-thick coronal sections evenly spaced by 90 μm were collected. Before each section was cut, a block-face photograph of the brain was taken (lateral resolution of 13 μm, EOS 5D Mark III, Canon). As block-face photographs were taken prior to tissue sectioning at the exact same position, the resulting images could be stacked to yield a 3D coherent brain volume for each subject that respects the geometry of the brains (Dubois et al., 2010). All tissue sections were immunohistochemically stained for Aβ-plaque detection with a 6E10 monoclonal antibody and a 3,3′-Diaminobenzidine revelation. Finally, stained tissue sections were digitized using a flatbed scanner (ImageScanner III, GE Healthcare) with a lateral resolution of 5 μm.

### 2.2. Image processing

**Figure 2:**
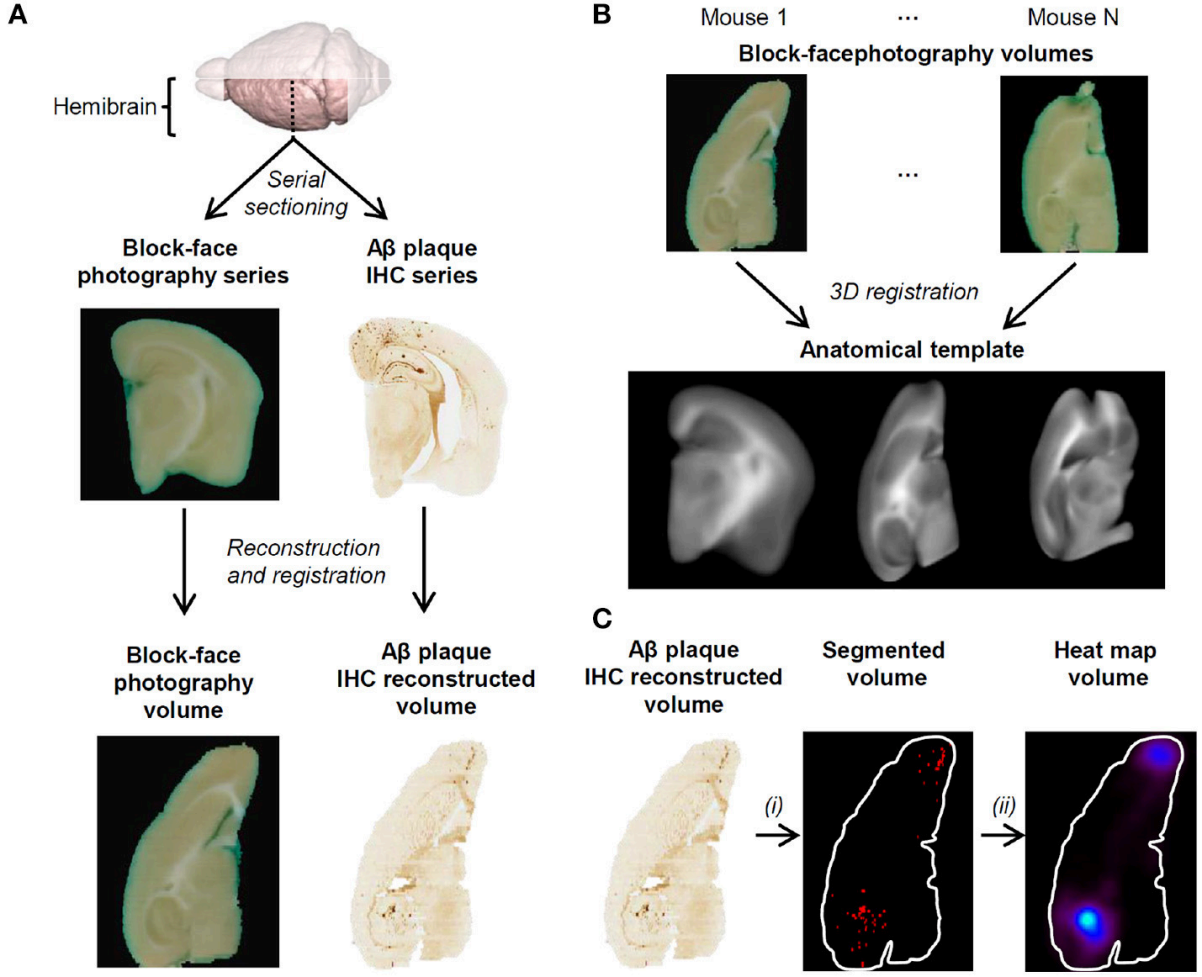
**(A)** 3D reconstruction of block-face photography and histology volumes. The block-face photography volume for each mouse is generated by stacking each photograph. Aβ plaque IHC stained coronal section images are stacked and registered to their corresponding block-face photographs. A reconstructed block-face photography volume and its corresponding 3D IHC volume are shown in the axial view. **(B)** The anatomical template is generated by registering every block-face photography volume onto a common reference. From left to right: coronal view, axial view and sagittal view of the anatomical template. **(C)** Aβ plaques are segmented on histology volumes using WRF (i), spatially normalized to the template and continuous-scale quantitative parametric map volumes are derived from the binary volumes (ii). All volumes are shown in the axial view at the same dorsoventral position.

This subsection describes the image processing steps that were performed prior to voxel-based inference on 3D histology images in the ADAM30 study (Figure 2). The image processing steps were implemented in the BrainVISA neuroimaging software (http://brainvisa.info).

#### 2.2.1. 3D reconstruction

For each individual, 3D reconstruction of block-face photographs and histology images was performed as described previously (Dubois et al., 2007). Briefly, block-face photographs were first segmented with an automatic threshold operation to extract the tissue from the background and then stacked in order to yield a 3D reference for each brain. IHC-stained section images were stacked and each individual image was registered to its corresponding block-face photograph by estimating a rigid transformation (translation, rotation) and an affine transformation (translation, rotation, scaling, shearing) that maximized the correlation coefficient between the images using the Block-Matching algorithm with three resolution levels (Dauguet et al., 2007). This allowed to reconstruct a coherent Aβ plaques IHC-stained volume for each individual and to correct for deformations due to histological procedures. Non-linear registration was not carried-out because the tissue embedding enabled to limit deformations within sections and because Aβ plaques IHC-stained volumes carry little neuroanatomical information to guide non-linear registration.

#### 2.2.2. Template generation

An average anatomical template was generated by, first, registering all the block-face photography volumes onto one chosen reference block-face photography volume and then by calculating the mean image from the registered block-face photography volumes.

3D registration between each block-face photography volume and the reference block-face photography volume was performed using a 3-step procedure validated previously (Lebenberg et al., 2010). A global rigid transformation was estimated based on the mutual information similarity criterion and Powell's optimization method (Viola and Wells, 1995), and then, an affine registration initialized with the rigid transformation was performed with the Block-Matching technique (Ourselin et al., 2001; Dauguet et al., 2007). Finally, a non-linear transformation was computed using the Free Form Deformation algorithm to improve registration locally by maximizing the mutual information between the images using the Broyden-Fletcher-Goldfarb-Shanno (BFGS) optimizer and a grid of 10 × 10 × 10 regularly spaced control points (Mattes et al., 2003; Lebenberg et al., 2010).

#### 2.2.3. Aβ plaque segmentation and registration

Aβ plaques were segmented using a weighted Random Forest model (WRF) (Breiman, 2001) with color and texture features as described and validated previously (Vandenberghe et al., 2015). This approach relies on a learning step which models ground-truth expert segmentation and a voxel-level classification step to segment images. The ground-truth dataset was constructed by extracting a set of 10 representative small 100 × 100-pixel-image patches from Aβ IHC volumes and subsequently manually segmenting them into 3 classes: background, non-stained tissue and Aβ plaques. In the learning step, 100 trees were constructed. As Aβ plaques represent only a very small fraction of the images compared to background and non-stained tissue, this class was weighted to increase its importance during the tree building process. The Aβ plaque class weight was tuned by leave-one-image-out cross-validation as described previously (Vandenberghe et al., 2015). In the classification step, each voxel was assigned to a class by a weighted majority voting of the classification trees. Each Aβ plaque IHC volume was fully segmented and a binary volume with segmented Aβ plaques was generated for each mouse brain.

Finally, high-resolution segmented volumes were all put into the same spatial referential by registering them to the anatomical template. As each segmented volume is in the same referential as its corresponding block-face photography volume, the registration was estimated between each block-face photography volume and the anatomical template using the aforementioned 3D registration approach and then the transformation was applied to each segmented volume. Voxel values in the registered segmented volumes were obtained by nearest-neighbor interpolation, therefore preserving the binary nature of the images.

### 2.3. Voxel-based analysis

In this subsection, we describe parametric map generation from binary images and cluster inference.

#### 2.3.1. Parametric map generation

In the simulation study, we investigated the performance of 3 techniques to generate paramatric maps which locally estimate the marker load: image binning; Voronoi tessellation (Barr and Schoenberg, 2010) combined with binning; Gaussian kernel smoothing combined with image binning. As the expected marker load is known in simulation images, the performance of each technique was evaluated by computing the mean squared error between the parametric map sample mean and the true expected marker load. The results from the simulation study enabled to choose the best method to be applied in the ADAM30 study.

Voronoi tessellation has been previously applied to estimate neuronal density in histology images (Duyckaerts et al., 1994) and does not require any parameter to be fixed. In contrast, image binning requires to specify a binning window-size and Gaussian-kernel smoothing requires to specify the Gaussian-kernel standard deviation. Fixing the size of the Gaussian-kernel standard deviation is particularly important as the bias and variance of the marker load estimation directly depend on it. The Gaussian-kernel standard deviation could be selected visually but, here, we propose a more rational approach to select an optimal kernel standard deviation. The optimal kernel standard deviation was selected based on bootstrap error minimization. First, a large number of bootstrap samples were generated by sampling individuals with replacement. For each bootstrap sample, segmented images were smoothed with a Gaussian kernel and the left-out individuals were used as a validation set. The bootstrap error (Hastie et al., 2009) was calculated as follows:

(2) $$\begin{array}{r} {\hat{Err}}_{boot}=\frac{1}{T}\overset{T}{\underset{j=1}{\sum }}\frac{1}{\left|C_{-j}\right|}\underset{b\in C_{-j}}{\sum }\frac{1}{n}\overset{n}{\underset{i=1}{\sum }}{\left({\hat{\mu }}_{x_{i}}^{b}-M_{j}\left(x_{i}\right)\right)}^{2}, \end{array}$$

where *T* is the number of individuals in the original sample, *C*_−*j*_ is the subset of bootstrap samples that do not contain individual *j* and |*C*_−*j*_| is the number of such samples, *n* is the number of voxels in the brain, *M*_*j*_(*x*) is the segmented binary image for individual *j* and ${\hat{\mu }}_{x}^{b}$ is the sample mean of smoothed images obtained from boostrap sample *b*. The bootstrap error was calculated for each experimental group separately. The optimal Gaussian kernel standard deviation is the one minimizing the mean bootstrap error.

Image binning was performed to explicitly reduce the image resolution and limit the total number of voxels in parametric maps. In the simulation dataset, the 2D window size was set to 18 × 18. For the 3D histology dataset, the binning window size was set to 18 × 18 × 1 voxels which provided low resolution parametric maps at an isotropic voxel-size of 90 μm. This choice for the parametric map voxel-size corresponds to the smallest cluster-size that would be deemed biologically relevant.

#### 2.3.2. Cluster inference

A voxel-based two-tailed Student's *t*-test was computed to compare the marker load between groups of individuals at each coordinate and the resulting *p*-value map was thresholded at a level *p* < 0.05. The Python scipy library implementation of the *t*-test was used throughout the study (https://www.scipy.org).

In the simulation study, F1 scores were calculated between inferred and true clusters using the number of true positives (TP), false positives (FP) and false negatives (FN):

(3) $$\begin{array}{r} F1=2TP/\left(2TP+FP+FN\right). \end{array}$$

Although parametric map generation greatly reduces the original size of 3D IHC volumes, voxel-based analysis throughout the brain involves a large number of statistical tests which raises the risk of false positive results. Therefore, in the ADAM30 study, a correction for multiple comparisons was performed using non-parametric cluster mass inference. Compared to the Bonferroni correction or the False Discovery Rate (Genovese et al., 2002), correcting multiple comparisons via cluster mass inference is advantageous for 3D IHC imaging as it does not rely on the total number of voxels in the brain and thus has the advantage of being insensitive to the binning value used for generating parametric maps. Cluster-mass was computed as the sum of the T-statistic values in the cluster (Bullmore et al., 1999). The distribution under the null hypothesis was constructed by performing 100 permutations on the individuals and recording the mass of the largest cluster from each permutation (Nichols, 2012). Finally, clusters that were larger than a threshold determined from the null-hypothesis distribution at *p* < 0.05 were considered as statistically significant.

Anatomical localization of the clusters was performed by registering the MICe digital mouse brain atlas on the anatomical template (http://www.mouseimaging.ca) (Dorr et al., 2008) following a protocol described and validated in Lebenberg et al. (2010) and further refined by referring to the Paxinos mouse brain atlas (Paxinos and Franklin, 2001).

### 2.4. Statistical analysis of Aβ plaque count and size

In the ADAM30 study, the effect of ADAM30 over-expression on the Aβ load was further investigated within the inferred clusters. For each mouse, the number and the size of Aβ plaques lying within clusters were recorded. Aβ plaques were defined on high-resolution segmented 3D IHC images as groups of connected Aβ plaque voxels in the coronal plane. Within cluster Aβ plaque number difference was tested with a two-tailed Students's *t*-test. Within cluster Aβ plaque size difference was assessed with a hierarchical linear model with mouse as a random factor and group as a fixed factor. Statistical tests were performed in R (http://cran.r-project.org) with the *stats* and *nlme* packages.

## 3. Results

### 3.1. Region-based analysis fails to anatomically finely characterize the effect of ADAM30 on the Aβ plaque load

In APP_*Swe, Ind*_ mice, Aβ plaques are most frequently observed in the cerebral cortex and the hippocampal region. In a previous study, we employed an region-based approach to show that ADAM30 reduces Aβ load in the hippocampal region and more globally, in the forebrain (including the hippocampal region and cerebral cortex) (Letronne et al., 2016). Table 1 shows the statistical comparisons between APP_*Swe, Ind*_-Cre-ADAM30 mice and APP_*Swe, Ind*_-ADAM30 in the hippocampal region and the cerebral cortex. A statistically significant effect was detected in the hippocampal region but not in the cerebral cortex.

**Table 1:** Comparative statistics between APP_*Swe, Ind*_-ADAM30 and APP_*Swe, Ind*_-Cre-ADAM30 using the region-based analysis.

**ROI**	**APP_*Swe, Ind*_-ADAM30**	**APP_*Swe, Ind*_-Cre-ADAM30**
Cerebral cortex	0.51 ± 0.46	0.27 ± 0.09
Hippocampal region	2.63^*^± 0.58	2.08 ± 0.42

*Data is shown as mean Aβ load ± SD*.

* *p < 0.05*,

*APP_Swe, Ind_-ADAM30 mice vs. APP_Swe, Ind_-Cre-ADAM30 mice (t-test)*.

### 3.2. Simulation experiments validate voxel-based analysis methodology

We investigated the best of the three candidate methods to generate parametric maps that can accurately synthesize 3D high resolution images to enable voxel-based analysis of 3D IHC imaging.

**Figure 3:**
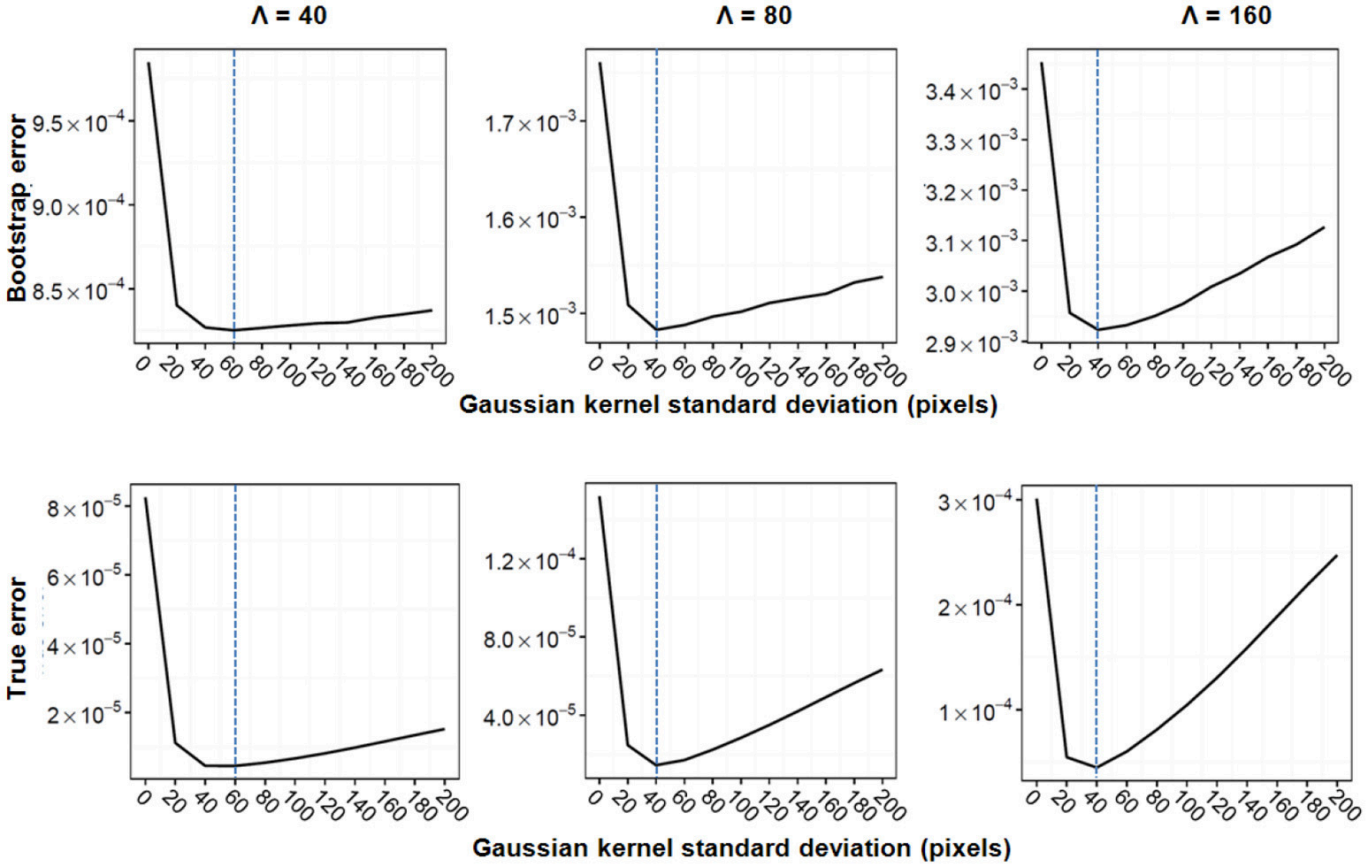
Gaussian kernel standard deviation selection for the three simulation experiments. Bootstrap error **(Top)** and true error **(Bottom)** are shown for the exacerbated group. From left to right: Λ = 40, Λ = 80, Λ = 160. The bootstrap error was calculated using 1000 bootstrap samples. Ten Gaussian kernel sizes were tested, minima are indicated with a dashed blue line.

**Figure 4:**
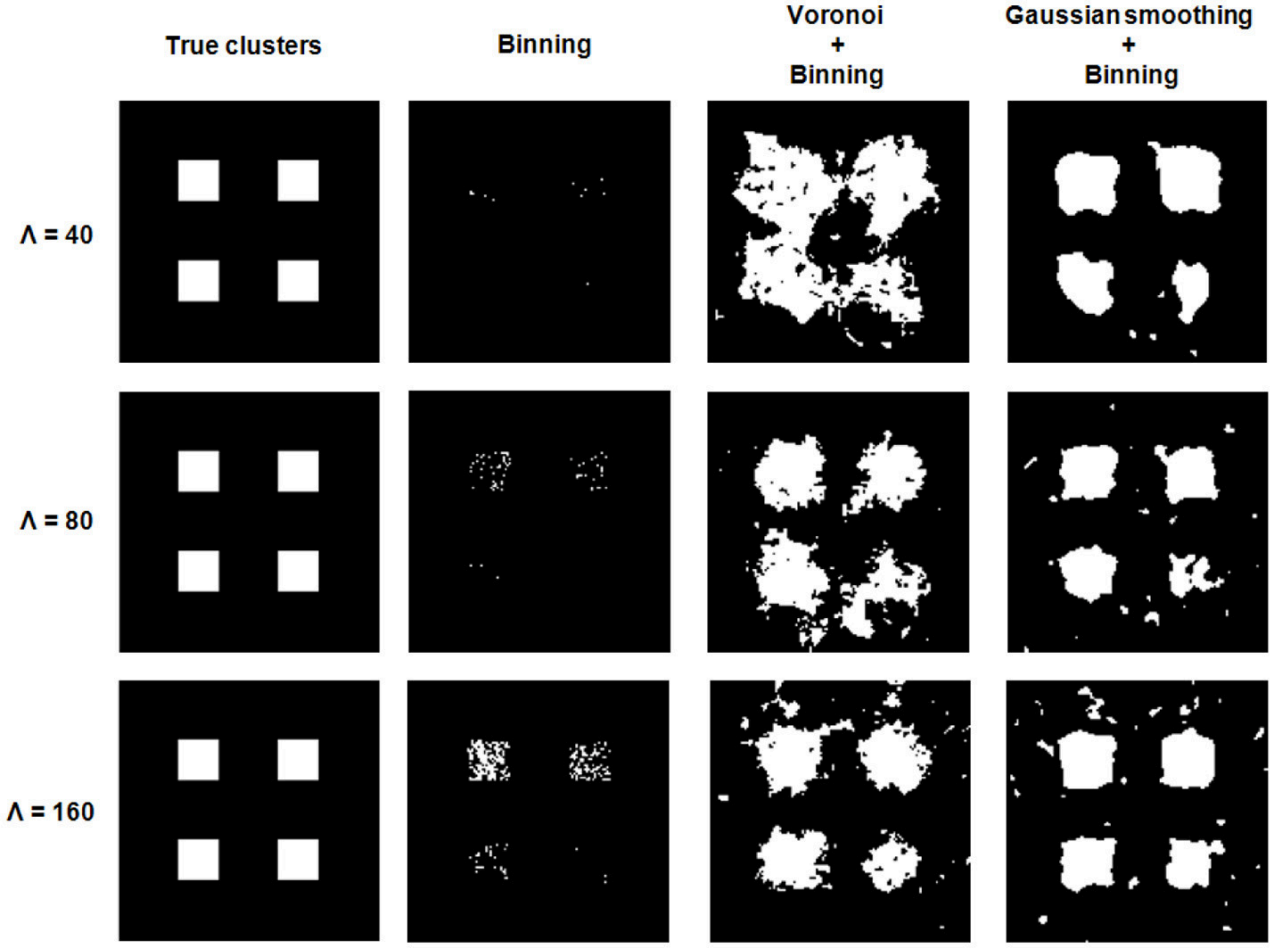
Cluster detection for the three simulation experiments. Each experiment consists of 10 images from the control group and 10 images from the exarcerbated group. From left to right: Location of the true clusters, clusters detected via binning alone, Voronoi tesselation combined with binning and Gaussian smoothing combined with binning.

Figure 3 illustrates Gaussian kernel-size selection using the bootstrap error. For all three experiments, the bootstrap error correctly pinpointed to the Gaussian kernel size which minimizes the true error indicating that the bootstrap error may be used to automatically select the optimal Gaussian kernel smoothing when the expected marker load is not known. Pixel-based Student's *t*-tests were performed between sets of parametric maps for each experiment and for the three approaches (image binning alone, Voronoi tessellation followed by binning and Gaussian kernel smoothing followed by binning). The three approaches resulted in very different marker load estimations and cluster detection results (Figure 4). Binning alone provided noisy marker load estimation and it barely allowed detection of differences between groups except for the experiment with the highest disk density (Λ = 160). While the Voronoi estimator allowed to detect clusters, their shapes were not recovered properly and, overall, the cluster sizes were overestimated as a result of imprecise load estimation in the neighborhood of the clusters. The Gaussian kernel estimation yielded smooth marker load estimation and the cluster shapes were recovered. Across all three experiments, Gaussian kernel smoothing followed by image binning provided parametric maps with the smallest true error compared the true marker load and allowed to detect clusters with the highest F1 score compared with the true clusters (Table 2). This approach was applied to the 3D histology study.

**Table 2:** Squared errors between the estimated marker load and the true population marker load as well as cluster detection F1 scores are shown for each simulation experiment and for each marker load estimation technique.

**Method**	**True error**	**F1**
Λ = 40		
Binning alone	8.26 × 10^−5^	0.13
Voronoi + Binning	8.27 × 10^−6^	0.44
Gaussian smoothing + Binning	**4.25×10^−6^**	**0.74**
Λ = 80		
Binning alone	1.52 × 10^−4^	0.07
Voronoi + Binning	2.67 × 10^−5^	0.55
Gaussian smoothing + Binning	**1.44×10^−5^**	**0.76**
Λ = 160		
Binning alone	3.01 × 10^−4^	0.37
Voronoi + Binning	8.14 × 10^−5^	0.64
Gaussian smoothing + Binning	**4.48×10^−5^**	**0.73**

*Best scores appear in boldface*.

### 3.3. Voxel-based analysis unveils the effect of ADAM30 in sub-regions of the hippocampus and of the cerebral cortex

**Figure 5:**
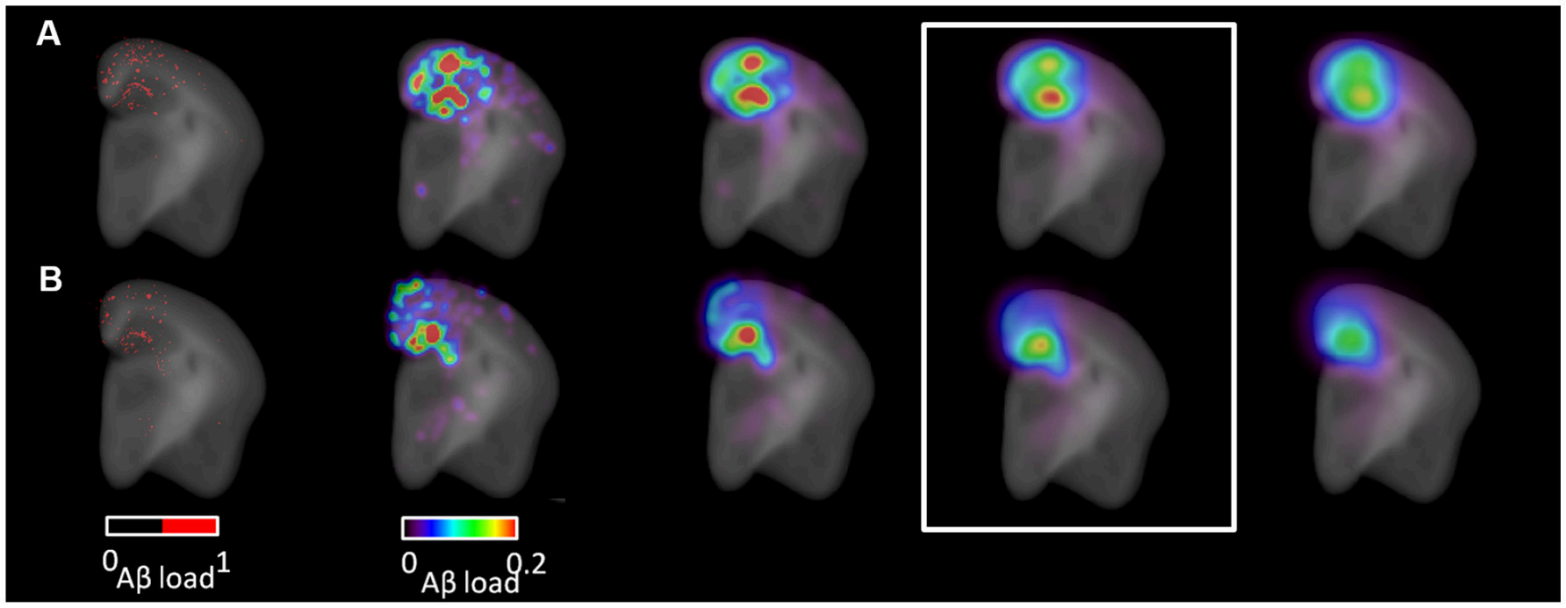
Parametric map generation. Coronal view images from **(A)** an APP_*Swe, Ind*_-ADAM30 mouse and from **(B)** an APP_*Swe, Ind*_-Cre-ADAM30 mouse. Left: segmented Aβ plaques (red) superimposed with the anatomical template. Right: parametric maps derived from the segmented image and superimposed with the anatomical template. Parametric maps are shown with increasing values of Gaussian kernel smoothing standard deviation (from left to right: 90, 180, 270, 360 μm). The Gaussian kernel standard deviation of 270 μm was selected for voxel-based testing (white rectangle).

**Figure 6:**
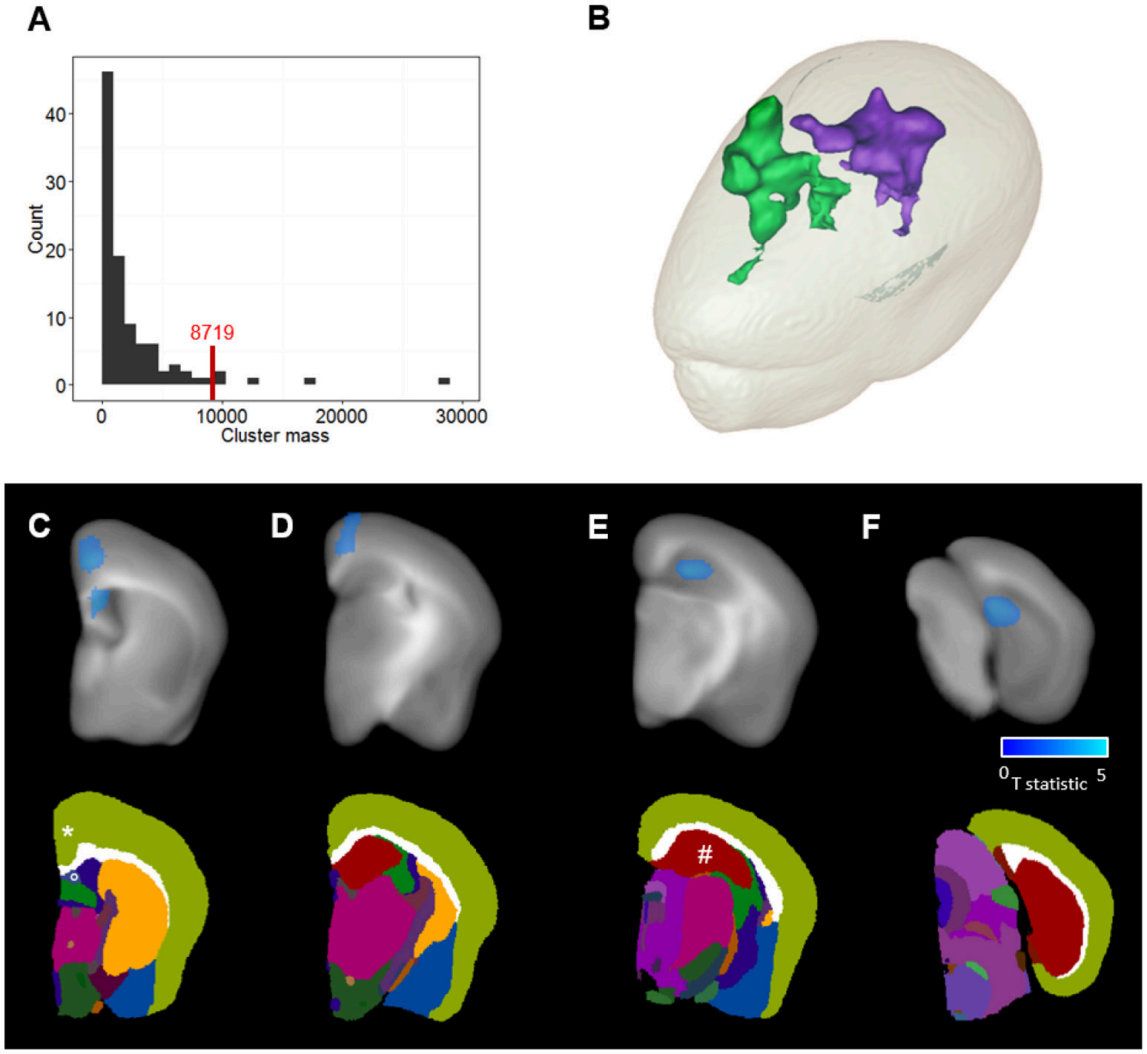
Clusters detection and anatomical localization. **(A)** Permutation-based cluster mass distribution under the null-hypothesis. The red line corresponds to the significance threshold at a risk α = 0.05. **(B)** 3D mesh representation of the two clusters where the Aβ load is reduced in APP_*Swe, Ind*_-Cre-ADAM30 mice compared to APP_*Swe, Ind*_-ADAM30. Green corresponds to the cortical cluster and purple corresponds to the hippocampal cluster. **(C–F)** Top: T statistic values within the clusters are shown in blue and overlaid with the anatomical template at selected coronal levels of the brain. Bottom: the MICe brain atlas at the same coronal levels. Clusters are localized in **(C)** the retrosplenial area of the cortex (*), the lateral septal nucleus (°), **(D)** the cingulate area of the cortex and **(E,F)** the dentate gyrus of the hippocampal region (#).

Following 3D IHC reconstruction (Figure 2) and parametric map generation (Figure 5), voxel-based analysis was carried-out in order to delineate more precisely the effect of ADAM30 over-expression in the brain of APP_*Swe, Ind*_ transgenic mice. Two significant clusters of reduced Aβ load were detected in APP_*Swe, Ind*_-Cre-ADAM30 mice compared to APP_*Swe, Ind*_-ADAM30 mice. This confirms the hypothesis that ADAM30 over-expression reduces the Aβ load (Figure 6). We did not detect any cluster of higher Aβ load in APP_*Swe, Ind*_-Cre-ADAM30 mice compared to APP_*Swe, Ind*_-ADAM30 mice.

The first cluster was identified in the lateral septal nucleus (Figure 6C) and the cerebral cortex (Figures 6C,D) while the second cluster was localized in the hippocampal region (Figures 6E,F). In the cerebral cortex, we could identify the cluster in the cingulate cortex areas 1 and 2 and the retropsplenial agranular cortex. In the hippocampal region we could identify the cluster in the dentate gyrus. The two detected clusters occupy 1.8% of the volume of the cerebral cortex and 7.1% of the volume of the hippocampal region.

### 3.4. Within-cluster investigation of Aβ plaque size and number suggests different mechanisms of actions in the cerebral cortex and hippocampus

**Figure 7:**
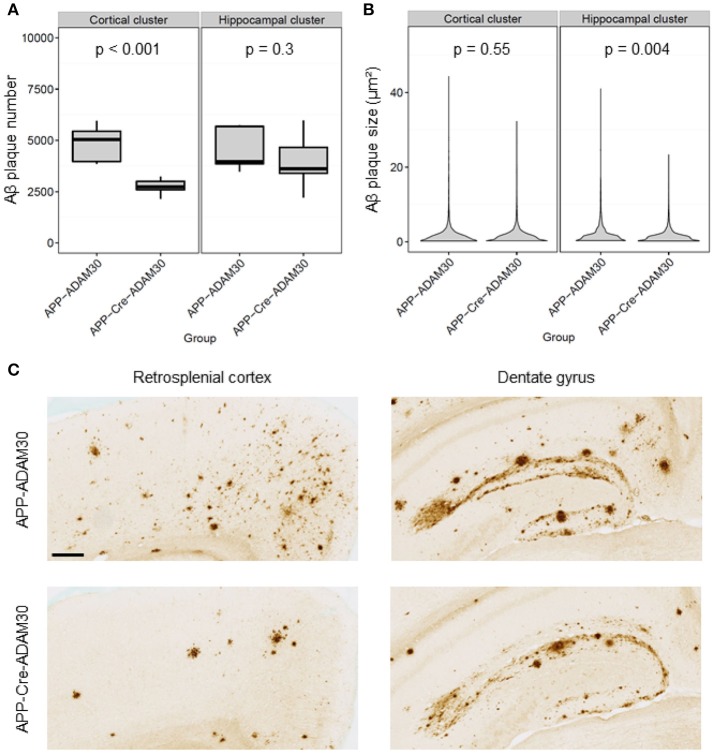
Comparisons of Aβ plaque number and size between APP_*Swe, Ind*_-ADAM30 and APP_*Swe, Ind*_-Cre-ADAM30 mice. **(A)** Boxplot with Aβ plaque number within the cortical and the hippocampal clusters. **(B)** Violin plot showing the distribution of Aβ plaques size within the cortical and the hippocampal clusters. **(C)** Zooms in 6E10 stained-tissue section images in the retrosplenial cortex and the dentate gyrus for both groups (scale bar: 200 μm).

After detecting clusters of significantly reduced Aβ load, we investigated if these differences were due to a difference in the number and/or the size of Aβ plaques between the groups. The Aβ plaque number was significantly reduced in the cortical cluster in APP_*Swe, Ind*_-Cre-ADAM30 mice (48 % drop in the mean number of Aβ plaques, Student's *t*-test, *p* < 0.001, Figure 7A). It remained similar in the hippocampal cluster for both groups (6% drop in the mean number of Aβ plaques in APP_*Swe, Ind*_-Cre-ADAM30 mice, Student's *t*-test, *p* = 0.3). Inversely, Aβ plaques had a reduced size in the hippocampal cluster in APP_*Swe, Ind*_-Cre-ADAM30 mice (hierarchical linear model, *p* = 0.004, Figure 7B) whereas we did not see any significant difference in the cortical cluster (hierarchical linear model, *p* = 0.55). Figure 7C shows zooms of the retrosplenial cortex and the dentate gyrus of APP_*Swe, Ind*_-ADAM30 and APP_*Swe, Ind*_-Cre-ADAM30 mice. Aβ plaque number reduction in APP_*Swe, Ind*_-Cre-ADAM30 mice is clearly apparent in the retrosplenial cortex but the few Aβ plaques observed in the APP_*Swe, Ind*_-Cre-ADAM30 mouse are rather large. On the other hand, in the dentate gyrus, an almost continuous band of Aβ plaques is apparent in the APP_*Swe, Ind*_-ADAM30 mouse while it seems thinner and more dislocated in the APP_*Swe, Ind*_-Cre-ADAM30 mouse.

## 4. Discussion

To our knowledge, the present study is the first to demonstrate the feasibility of voxel-based analysis in 3D imaging of immunostained brain tissue. A first challenge in performing voxel-based analysis of the rodent brain at the microscopic level is to produce the images. Specific challenges may arise depending on how the tissue sample is produced and digitized. Here, tissue sections were stained for Aβ plaques and then reconstructed in 3D thanks to block-face photography which served as a geometrical reference. 3D histology has demonstrated its potential for high-throughput production of whole-brain images of immunostained specimens so that group-studies including dozens of animals can be performed in reasonable time (Vandenberghe et al., 2016). Histology color images were segmented with a supervised classification approach based on color-texture features and WRF classification. This approach has been shown to be robust to various artifacts which arise during histology processing (non-specific staining, tissue folding, debris on the glass-slide) (Vandenberghe et al., 2015). Finally, segmented images were spatially normalized using block-face photography because it provides good anatomical contrasts and has been successfully used to construct anatomical templates in previous 3D autoradiography studies (Dubois et al., 2010; Lebenberg et al., 2010). In this work image processing steps were adapted for 3D histology, but if brain 3D images were to be acquired through light-sheet microscopy or reconstructed from optical tomography, the most appropriate way to segment and spatially normalize images may vary according to each imaging modality specificities.

A second challenge is that microscopy images are not naturally suited for voxel-based analysis. Indeed, it would not be realistic to attempt voxel-based inference directly at microscopic resolution because the inherent inter-individual variability of brain microstructure would overshadow inter-group variability. A crucial part in the proposed approach consists in segmenting the marker of interest at microscopic resolution and then in smoothing the segmented high-resolution images to provide accurate voxel-based estimation of the marker load at a lower resolution. For voxel-based analysis to be reliable, the parametric map sample mean for a given group should estimate the population mean as precisely as possible. Ideally, the smoothing should not excessively alter the original images (i.e., low bias estimation) and it should give reproducible estimates across individuals from the same group (i.e., low variance estimation). The optimal Gaussian kernel size may vary from one study to another according to, for instance, the density of the segmented objects in high-resolution images and the number of individuals included in the study. Here, we proposed a bootstrap error approach which can be used to determine the optimal Gaussian kernel size. In the simulation study, this approach predicted the value of the optimal kernel size in various experimental conditions and it allowed to approximate the shape of the clusters. Future studies are warranted to validate this approach under more varied conditions such as when object shape or size varies between groups. Smoothing the high-resolution segmented images results in a reduction of image resolution. Because neighboring voxel values are highly correlated, smoothed images can be binned to explicitly reduce the image size. This also provides two important practical advantages: it reduces the impact of small registration errors that can arise during spatial normalization and it limits the problem of multiple comparisons. Other methods could be used to estimate the marker load. For instance, a limitation of Gaussian kernel smoothing is that the amount of smoothing is constant. In order to preserve more details in very dense regions while smoothing sparser regions, an interesting perspective would be to use adaptive Gaussian smoothing (Abramson, 1982) to select the kernel size on a per voxel basis. Additional evidence is warranted to determine the most appropriate spatial smoothing approaches across various experimental conditions and markers of interest.

A third challenge which is common to preclinical voxel-based studies is the small sample-size compared to most voxel-based studies in humans. Small sample-size strongly hinders statistical power when performing corrections for multiple comparisons at the voxel-level. Voxel-based studies are often valuable in animals because effect-sizes are usually expected to be larger in than in humans due to for example, genetic engineering or the administration of high doses of active compounds. In addition, in preclinical studies populations are standardized according to strain, gender age and weight which usually enable to rely on smaller sample sizes than in humans. Here, to further mitigate the risk of type II errors, we used permutation-based cluster inference which takes advantage of the dependence between voxels.

The developed voxel-based analysis was applied to dissect the effect of ADAM30 over-expression on the Aβ plaque load. In a previous study, ADAM30 was shown to be a major actor in the metabolism of the Amyloid Precursor Protein which is necessary for the appearance of Aβ plaques (Letronne et al., 2016). Also, an region-based approach enabled to show that ADAM30 significantly reduces the Aβ plaque load in the hippocampus of APP_*Swe, Ind*_ transgenic mice. Here, voxel-based analysis enabled to precisely locate the effect of ADAM30 in APP_*Swe, Ind*_ mice in the cingulate cortex, the retrosplenial cortex, the lateral septal nucleus and the dentate gyrus. Interestingly, the cingulate and the retrosplenial areas of the cerebral cortex are primarily involved in cognitive and memory function and the dentate gyrus is responsible for spatial memory (Spreng et al., 2009; Vann et al., 2009) and these areas are among the first ones to undergo functional alterations in Alzheimer's disease (Lenartowicz and McIntosh, 2005; Pengas et al., 2010). Using a single region-based analysis, a significant effect of ADAM30 was found in the hippocampus and a weak trend was found in the cerebral cortex. Notably, voxel-based analysis detected clusters that span over 1.8% of the cerebral cortex and 7.1% of the hippocampal region. Therefore, in this study, the inability of the region-based approach to detect the ADAM30 effect in the cerebral cortex could be explained by the restricted spatial extension of this effect. A decisive advantage of the proposed voxel-based approach is its ability to detect local effects on the brain microstructure. However, if changes between groups are distributed over entire regions, the region-based analysis could be more powerful than the voxel-based approach.

Once clusters are detected, information from high-resolution images can be exploited to explain the differences observed at lower resolution. This is unlike circular analysis as the focus is to explain differences at the microscopic scale, not to confirm what has been already observed at the lower resolution. Here, we found that ADAM30 reduces Aβ plaque number in the cingulate and retrosplenial cortices while it reduces the Aβ plaque size in the dentate gyrus. The lower number of Aβ plaques in the cingulate and retrosplenial cortices of APP_*Swe, Ind*_-Cre-ADAM30 mice suggests that ADAM30 can prevent the formation of new Aβ plaques in these regions. Nonetheless, Aβ plaques have similar sizes in both groups which indicates that once seeded, Aβ plaque growth is not altered by ADAM30 over-expression. Contrastingly, in the dentate gyrus, the lower Aβ plaque size indicates that ADAM30 over-expression could slow the Aβ plaque growth in the dentate gyrus. Based on these results and the fact that clusters are located in functionally connected regions, it could be hypothesized that ADAM30 has an Aβ lowering effect in the hippocampal region which could then lead to a reduced spreading of Aβ plaques in tightly connected areas of the cerebral cortex. Notably, Aβ plaques are very clumped in the dentate gyrus which makes it hard to study them individually. Aβ plaques are pathological aggregates without a defined structure and several Aβ plaques in close proximity can grow until they form a unique structure. Thus, studying the Aβ plaque size and number in the dentate gyrus is inherently difficult and a definitive conclusion about the effect of ADAM30 on individual Aβ plaques formation and growth in the hippocampus requires additional experiments.

There are multiple ways which could enhance and refine the proposed methodology. The simulation study only considered scenarios where the density of objects varies between groups. It could be further expanded to include scenarios where shape or size of objects varies or combinations of thereof. One limitation of the 3D histology study is that the choice of a reference brain to use as template was somehow arbitrary. A large body of work in neuroimaging has been devoted to methods to generate templates in an objective manner (Kochunov et al., 2002; Lepore et al., 2008). Future work could be undertaken to apply such methodologies to generate templates for 3D immustained imaging. Another limitation is that we have only explore one way of inferring clusters. An interesting perspective would be to investigate adjusted cluster size to account for non-stationarity in the images (Salimi-Khorshidi et al., 2011). Besides, Random Field Theory as well as additional cluster-based inference approaches could be further explored. Finally, permutation tests with a greater number of permutations or using tail-fitting approaches could be further investigated (Winkler et al., 2016).

In conclusion, this work describes the first application of voxel-based statistical analysis to 3D imaging of immunostained tissue. Given the large content lying in microscopic images, this work opens new avenues for the quantitative analysis of brain microstructural adaptation to pathology.

## Author contributions

TD, J-CL, MD, FF, and PH contributed conception and design of the study. A-MA, FL, and A-SH performed experiments. MV, NS, ES, and YB developed the methodology. MV performed statistical analysis. MV wrote the manuscript. A-SH, TD, YB, FF, and MD contributed the manuscript. All authors read and approved the submitted version.

### Conflict of interest statement

The authors declare that the research was conducted in the absence of any commercial or financial relationships that could be construed as a potential conflict of interest.
